# Spatiotemporal historical datasets at micro-level for geocoded individuals in five Swedish parishes, 1813–1914

**DOI:** 10.1038/sdata.2017.46

**Published:** 2017-04-11

**Authors:** Finn Hedefalk, Patrick Svensson, Lars Harrie

**Affiliations:** 1 Department of Physical Geography and Ecosystem Science, Lund University, SE-220 07 Lund, Sweden; 2 Centre for Economic Demography and Department of Economic History, Lund University, SE-223 62 Lund, Sweden

**Keywords:** Geography, History, Interdisciplinary studies

## Abstract

This paper presents datasets that enable historical longitudinal studies of micro-level geographic factors in a rural setting. These types of datasets are new, as historical demography studies have generally failed to properly include the micro-level geographic factors. Our datasets describe the geography over five Swedish rural parishes, and by linking them to a longitudinal demographic database, we obtain a geocoded population (at the property unit level) for this area for the period 1813–1914. The population is a subset of the Scanian Economic Demographic Database (SEDD). The geographic information includes the following feature types: property units, wetlands, buildings, roads and railroads. The property units and wetlands are stored in object-lifeline time representations (information about creation, changes and ends of objects are recorded in time), whereas the other feature types are stored as snapshots in time. Thus, the datasets present one of the first opportunities to study historical spatio-temporal patterns at the micro-level.

## Background & Summary

Studying how micro-level geographic factors have influenced human living conditions over long time periods provides many new and important insights. However, in the field of historical demography studies have primarily been limited to the macro level due to a lack of individual-level and longitudinal geocoded historical databases to enable studies at the micro-level. Currently, only a few historical longitudinal demographic databases have geocoded individuals to more precise locations, such as streets or property units^1^. In most longitudinal historical populations, individuals are linked to a less precise position such as villages, parishes or other larger administrative units. Most historical demographic databases that are geocoded on a more detailed level (e.g., ref. 2) only cover a snapshot in time, which is incompatible with longitudinal analyses.

**Figure 1:**
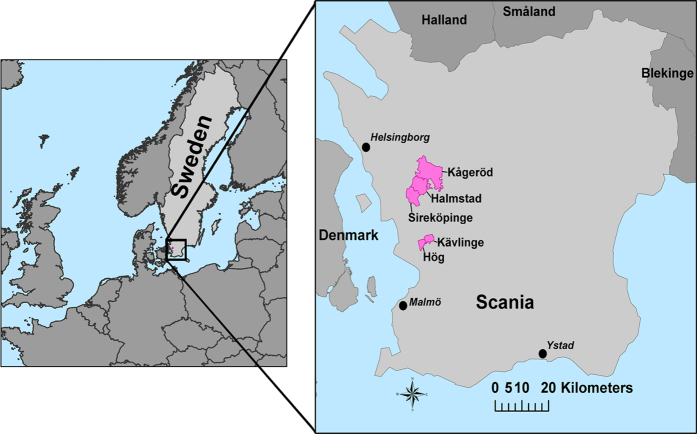
The five parishes (Hög, Kävlinge, Sireköpinge, Halmstad and Kågeröd) covered in the datasets.

The datasets presented here adds geographic information to the Scanian Economic Demographic Database (SEDD). The SEDD covers an area of five rural parishes (approximately 130 km^2^) in southern Sweden (Fig. 1). The SEDD includes detailed demographic and economic information from 1646 to the present and is the result of project collaborations between the Centre for Economic Demography (CED) and the Regional Archives in Lund^3^. The SEDD has been extensively used in historical research (e.g., refs 4–6). All individuals in the SEDD are followed from birth or in-migration to death or out-migration. The database contains continuous information on the family and household structure, and on dates of births, marriages, and deaths occurring in the parishes. Characteristics such as socioeconomic status, as well as causes of death, are known for the period 1813–1914. The primary sources for the SEDD are vital registers, catechetical examination registers and annual poll-tax registers. However, the SEDD population had not been previously geocoded on a detailed level. In a recent study, we geocoded a subset of SEDD with approximately 53,000 individuals for the time period 1813–1914 (ref. 7). This geocoding enabled the first longitudinal studies of geographic factors at the micro-level^8^.

Three types of datasets for the five rural parishes are provided in this paper: 1) property units (which the individuals in the SEDD are linked to); 2) historical wetlands; and 3) additional historical geographic data, such as infrastructure and buildings. The primary sources of the geographic data are historical maps and cadastral dossiers that encompass the five parishes for the period 1757–1914, as well as textual sources, such as annual poll-tax registers. The property units and wetlands are stored in temporal representations of longitudinal object-lifelines^9^. That is, for each property unit, we know when it was created, reformed, and ceased to exist; for the wetlands, we know when they were drained. The reason for storing wetlands in object-lifelines is to enable accurate proximity analyses for the whole study period. Proximity to wetlands and still water may indicate exposure to various diseases such as water-borne diseases and malaria (until the 20th century, malaria was a problem in Europe, and wetlands likely provided habitats for mosquitoes that transmit malaria^10^). Therefore, the possibility of estimating the exposure to wetlands is necessary for researchers to understand causes of mortality in historical societies. These wetlands were gradually drained during the 19th and 20th centuries; thus, their lifelines were estimated to enable estimations of dynamic geographic variables. Besides, the drainage of wetlands substantially changed the landscape and likely increased the agricultural productivity of the land. Thus, it is possible to study how these interventions affected the individual-level economic conditions of the farmers. Finally, the additional historical geographic data (roads, buildings and water) were digitized from several series of historical maps as temporal snapshots at single points in time. Consequently, we have annual information about the shape of each property unit, which enable us to accurately trace the residential histories of individuals in time and space. Combined with the historical geographic data and other modern geographic data, such as elevation and soil conditions, the datasets can be used for spatio-temporal analysis, as well as the inclusion of geographic factors in longitudinal analyses of historical demographic data. Thus, they present opportunities to address novel research questions not only in the fields of historical demography and economic and agrarian history but also in many other fields, such as epidemiology, medicine and geography.

## Methods

The creation of the datasets consisted of three process steps: (1) creating geographic snapshot data by georeferencing and digitizing historical maps; (2) transforming the snapshot property unit and wetland data into an object-lifeline representation using Supplementary data; and (3) linking individuals in the SEDD to the property units in which they lived. The digitized buildings, communications and streams remained in their snapshot representations.

### Data sources

**Figure 2:**
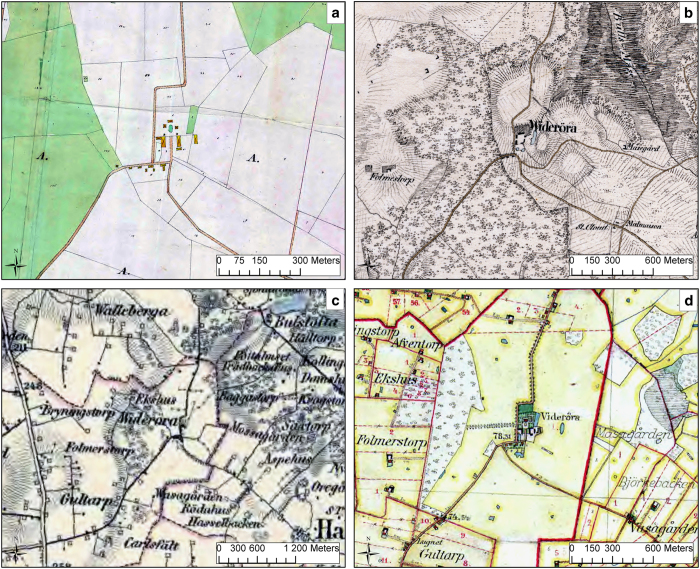
Four maps that show Videröra mansion at the border of Halmstad parish. (**a**) LSM from 1831. (**b**) MTSM from 1812–1820. (**c**) TM from 1860. (**d**) EM from 1910–1915. (Source: ref. 17).

The maps were obtained in digital format from the Lantmäteriet (the Swedish mapping, cadastral, and land registration authority). The historical maps originate from four map series: land survey maps (LSMs), military topographical survey maps (MTSMs), topographic maps (TMs), and economic maps (EMs). The 150 digitized cadastral dossiers (CDs) were primarily used to document changes in the property units and to create an object-lifeline representation. As shown in Table 1 and Fig. 2, the maps were created at different scales and for different purposes. The LSM and CD maps primarily focused on documenting juridical borders, such as property units, but also documented buildings and land use, communications and the natural landscape. The MTSM and TM were constructed for military purposes and thus, focused on the natural landscape, land use, topography, communications and physical objects, such as buildings. The EMs documented property unit borders and the natural landscape, land use, communications and buildings.

For supplemental textual data, we used the Swedish poll-tax registers that cover the study period, which contain the addresses, taxation value (of the property unit), and household and owner information of each person who had to pay taxes. These individual taxation values can be used to detect possible geometric changes in the property units and estimate the lifelines of the property units.

### Georeferencing and digitizing historical maps

We georeferenced and digitized the historical maps and cadastral dossiers in Table 1. In the georeferencing process, we used orthophotos from 1940 and modern topographic web maps to identify common points in the historical maps, such as churches, terrain features, crossroads, and river junctions. The historical maps were transformed using a piecewise interpolation method (spline). Geographic objects, such as property units, buildings, roads, railroads and streams, were digitized as temporal snapshots from the maps. The georeferencing and digitalization steps were performed using ArcGIS Desktop 10.3 (http://www.esri.com/software/arcgis/arcgis-for-desktop). Historical and digitized wetlands from the MTSM series were received from the Swedish County Administrative Board of Skane.

### Creating object-lifeline representation of the property units and geocoding individuals

In this step, we transformed the snapshot data into an object-lifeline model and linked individuals to the property units in which they lived. This method is described in ref. 7.

To create an object life-line representation of the property units we combined the digitized property units from the LSMs, CDs, and EMs with information from the poll-tax registers and cadastral dossiers. With this information, we learned when the property units were created, changed, and ceased to exist. The main aim of the geocoding was to establish links between the records in the poll-tax registers and the digitized property units, which was simultaneously performed with the estimation of the property units’ lifelines.

For the geocoding, we primarily used information from the Swedish poll-tax registers, which contains the addresses (property units) of each person who had to pay taxes. The Swedish poll-tax registers are on a household level, which indicates that only the head of the household is noted with a full name. However, each individual in the SEDD is linked to the families and households to which they belong, and most of these families and households have a correspondence in the poll-tax registers. In this project, we used a subset of the SEDD from 1813 to 1914, which contains approximately 53,000 individuals, 10,000 households and 184,500 annual poll-tax records, in which 90.4% of the individuals experiencing an event in the demographic data are linked to the poll-tax registers (96.2% for the period 1829–1914 when continuous population registers exist for all five parishes). The subset contains all the individuals living in the five parishes for the period 1813–1914. We began the geocoding in 1,813 as information about the individuals’ migration within and between parishes from this year and onwards is available. We stopped in 1914 due to personal integrity constraints.

**Figure 3:**
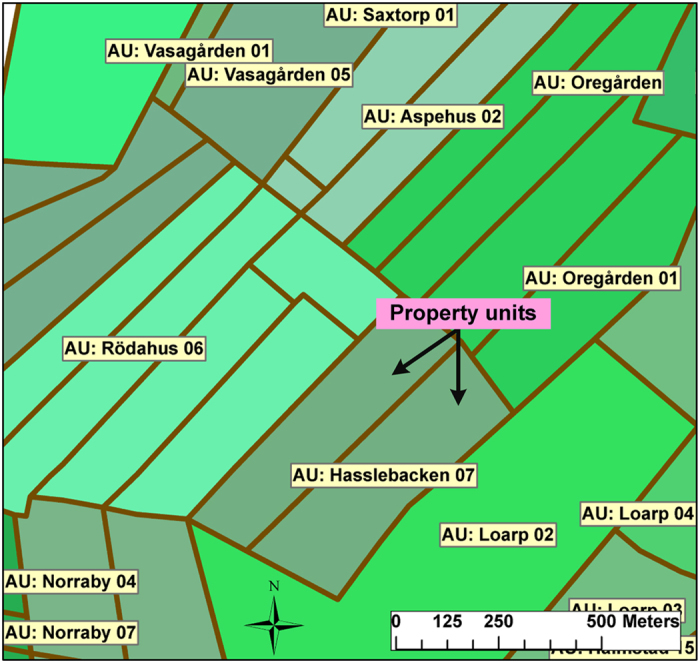
Property units and address units in 1871 in Halmstad parish. Property units that share an address (identical colour in the map) constitute one address unit. For example, the two property units with the address Hasslebacken 07 constitute one address unit. Only the address units are labelled.

**Figure 4:**
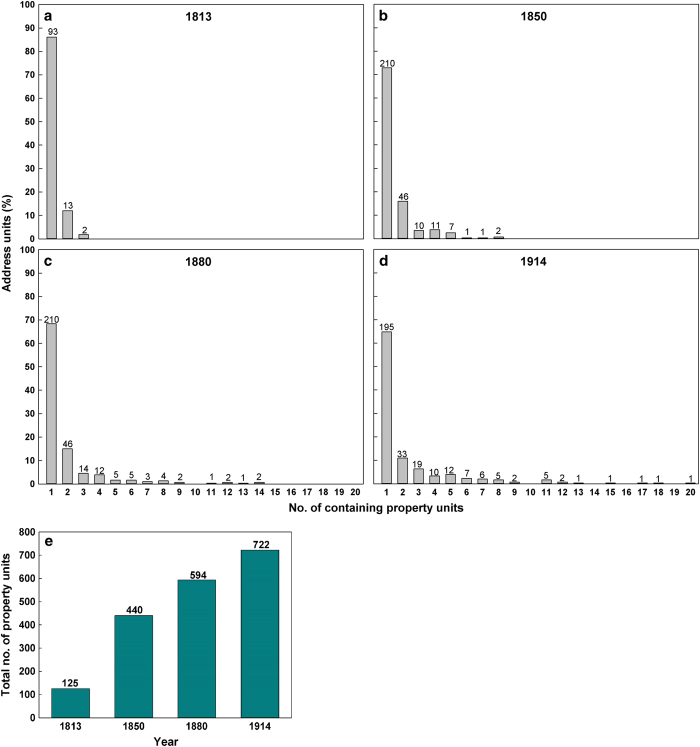
Changes of address units and property units for the period 1813–1914. (**a**–**d**) Percentage of address units constituted by a given number of property units for the years 1813, 1850, 1880 and 1914; the values on the bars represent number of address units with the specific amount of property units. (**e**) Total number of property units for the years 1813, 1850, 1880 and 1914.

The geocoding and creation of object-lifelines required extensive investigation for several reasons. For instance, before the land reforms (conducted between 1757 and 1849 in the parishes), all individuals lived in small villages and cultivated nearby scattered plots. After the land reforms, the self-owned farmers received a cohesive piece of land, to which they also moved. We denote these lands as *property units* (Fig. 3). Although the property units were usually devoted to agriculture, some of the units also contained forest lands. Throughout the study period, several of the property units were subdivided or partitioned into smaller units (consistent with rapid population growth). However, the property units did not receive new addresses; thus, multiple property units often share addresses. We denote the set of such units as an *address unit* (Fig. 3). With some exceptions, they are located close and adjacent to each other. The number of property units per address unit, as well as the total number of property units, was constantly increasing throughout the study period (cf. Fig. 4).

The geocoding on the address unit level was more straightforward as the poll-tax register contains annual information about the address unit for the head of the family. However, we also aimed to geocode on the property unit level. To mitigate the problem of units that share addresses, we used taxation values in the poll-tax registers combined with textual sources in the maps and cadastral dossiers to separate the units that share addresses. We also traced the owner histories of the property units when property units shared both taxation values and addresses^7^. For several records, extensive manual research was required to achieve a substantial number of reliable links.

### Creating object-lifeline representation of wetlands

To create an object-lifeline representation of the wetlands in the study area, we combined information about 1) digitized historical wetlands from the MTSMs from 1812–1820 (provided by the Swedish County Administrative Board of Skane); 2) modern wetland and water data; 3) soil type data; 4) the EM from 1910–1915; and 5) data regarding registered joint drainage units before 1920 within the five parishes.

For each digitized wetland from the 1812–1820 MTSMs, the end date was estimated using overlapping joint drainage units. These drainage units were joint initiatives by several property unit owners with the purpose of draining a specific area and sharing the cost. These drained areas were registered during the 19th century and have been scanned and digitized^11^. Thus, a drainage unit that covers a wetland indicates that the wetland was drained. Observations of wetlands and water bodies in the EMs from 1910–1915 were also compared with the digitized wetlands, the modern digitized wetlands and other water bodies. A soil type map^12^, in which soil types such as peat and muddy sediments indicate historical wet areas, was also used.

Because of the uncertainty in several of the start and end dates of the wetlands, we also used intervals representing the minimum and maximum start and end date. For example, if a digitized wetland from the 1812–1820 MTSM was not observed in the 1910–1915 EM nor had any overlapping joint drainage unit, its maximum end date was set to 1909. Moreover, if a wetland was observed as being more similar in shape and size to a modern wetland, the maximum start date of the digitized modern wetland was set to 1910.

**Figure 5:**
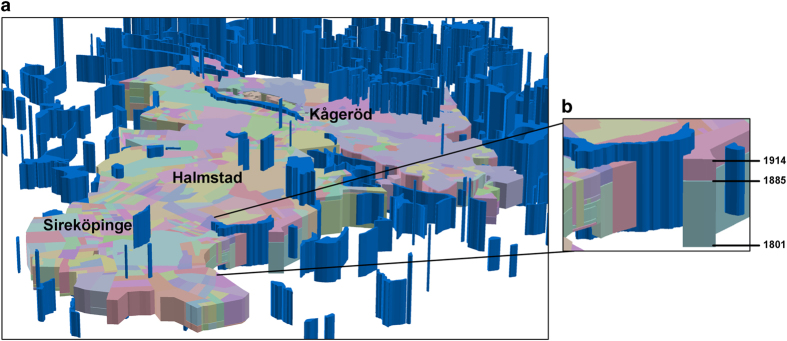
Property units and wetlands (blue) in object-lifeline representations. (**a**) The parishes Sireköpinge, Halmstad and Kågeröd (**b**) Enlarged area of some property units (including Bångstorp 01).

Figure 5 shows the property units and wetlands in the object-lifeline representation of the parishes Sireköpinge, Halmstad and Kågeröd. In the figure, the temporal dimension is displayed on the *z*-axis, and the height of each object represents its existence in time. Note that several wetlands are not visible in the figure because they are covered by property units (i.e., they disappeared before 1914). Figure 5b shows an example of the property unit Bångstorp 01, which existed for the period 1801–1885. Thereafter, it was subdivided, and a new property unit (also with the address Bångstorp 01) was created in 1885. The maximum end time of the property units is 1914, whereas the maximum end time for the wetlands is 2007.

## Data Records

The data records contain eight datasets on ESRI Shapefile format (Table 2); each contains four files with the extensions.shp,.dbf,.shx, and.prj. The datasets are stored in the Harvard Dataverse (Data Citation 1). The spatial reference for all geographic data is the official Swedish geodetic reference system Sweref 99 TM (EPSG: 3,006), which is a UTM 33N projection of the Swedish realization of the European Terrestrial Reference System 1989 (ETRS89). The attributes for each of the datasets are described in Tables 3–7. Empty cell values in the datasets represent missing information. In addition to the information in Tables 3–7, the data records contain a codebook (Codebook_SEDDGeo.pdf) that lists the classifications used in the categorical attributes.

### Links to the scanian economic demographic database (SEDD)

The geocoded demographic information from the SEDD is freely obtained from http://www.ed.lu.se/databases/sedd/sedd-public-access^3^.

To access the dataset, the users need to send an e-mail request to the Centre for Economic Demography (for reporting data use). The SEDD dataset is longitudinal and contains individual event information on the total population within the five parishes for the period 1813–1914 (cf. ref. 13). The structure of the dataset is a table in which each row contains the identifier of one unique individual and supplied attributes of the individual. The table also contains events of specific occurrences, such as birth, marriage, outmigration and death. Each individual has one or more episodes, which is the period of time when all variables connected to the individual are constant. These episodes are defined by start- and end-dates. Each time a variable changes a value; e.g., if a person moves to another household, a new row with a new episode is included in the table. The links to the geographic data are specified in the *PropID* attribute, and changes each time the individual is moving to another location. In addition, the *PropLevel* attribute specifies whether the links are on address or property unit level. The links in the geographic data are specified by the attributes *puId* and *auId* in the Property Units dataset. By using these link attributes in combination with date we can easily link individuals to the digitized property units, as well as link geographic context variables to the individuals in the SEDD.

## Technical Validation

### Positional accuracy

The evaluation of the positional accuracy of the digitized property units is based on a subset (7.5%) of all property units. The selection was made based on the criteria that the property unit borders have not changed until now. This selection enables us to use modern property unit borders obtained from the Lantmäteriet (the Swedish mapping, cadastral, and land registration authority) as reference data. This subset should be a representative selection in terms of positional accuracy. In the evaluation, two measures of positional accuracy are used.

The first measure of positional accuracy is based on the buffer-overlay-statistics (BOS)^14^ method. The basic idea with this method is to create buffers around a line to be evaluated and another buffer around a reference line, and then base the quality evaluation on intersections between the buffers (and their set complement). In our case, we created buffers around the historical property unit boundaries (*HB*) as well as buffers around the modern boundaries (*RB*). Then the following measures were estimated:

(1)QBOS=Area(HB∩RB)Area(HB∩RB)+Area(HBRB)+Area(RBHB)

(2)QA=Area(HBRB)Area(HB∩RB)+Area(HBRB)+Area(RBHB)

(3)QB=Area(RBHB)Area(HB∩RB)+Area(HBRB)+Area(RBHB)

**Figure 6:**
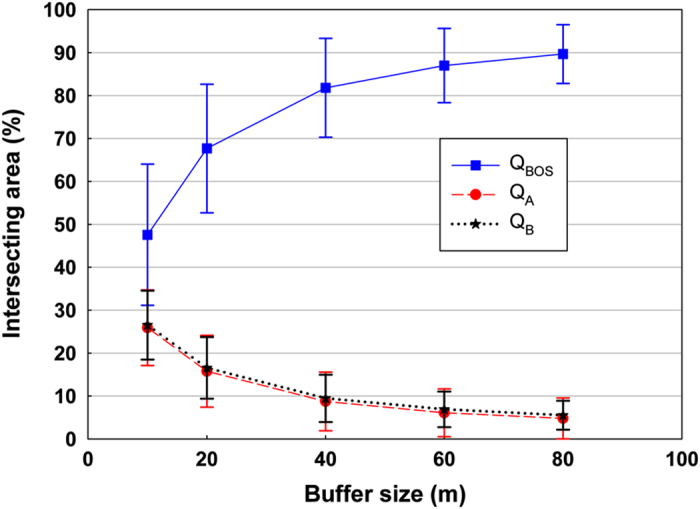
Mean BOS accuracy results for the historical property units (HB) compared to the modern property units (RB). The error bars represent the s.d.

That is, *Q*_*A*_ is defined as the relative complement of *RB* in *HB;* and *Q*_*B*_ is defined as the relative complement of *HB* in *RB.* The quality parameters are computed for a set of buffer sizes to provide the buffer overlay statistics (Fig. 6). A high positional accuracy is indicated by high *Q*_BOS_ values and low Q_A_ and Q_B_ values. When using a buffer size of 80 meters, we include approximately 90% of the reference boundary lines.

The second measure for estimating the positional accuracy is based on the Euclidean distance between the historical property unit centroid and the centroid of the corresponding modern property unit. The non-normal error distribution of the property unit centroids may limit some of the usefulness of the statistics used in Table 8, such as s.d., mean, and the root mean square error (RMSE). The 95% RMSE was calculated after removing 5% of the outliers, which is a more representative measure for non-normal distributions^15^.

Based on the result of the two quality measures, we conclude that the positional accuracy of the property unit centroids is approximately 10–15 meters, whereas the accuracy for the property unit boundaries is slightly lower.

It was more difficult to determine the positional accuracy of the wetlands. The RMSE of the georeferenced 1812–1820 MTSMs, from which most of the wetlands had been digitized, was estimated to be 30 meters. This estimation is based on a comparison of unchanged objects, such as churches, crossroads and residential buildings in the geocoded MTSM, with the reference data World Imagery web map^16^ (geometrical resolution between 30–60 cm). Apart from the geometric uncertainty in these well-defined unchanged objects, there are additional uncertainties in the mapping of wetlands; for example it is difficult to define borders of wetlands and also to define what a wetland is.

### Match rate and geocoding level

Match rates for the geocoding on the property unit and address levels for the five parishes are presented in Table 9. The rate is measured in percent person-years for the individuals in SEDD. The geocoding period is split into two periods to show the differences in the linking levels before and after the land reforms had been conducted within all parishes. Note that the geocoding match rate has been updated and improved in this paper compared with a previous study^7^.

### Spatio-temporal topology validation (verification of overlapping polygons in space and time and verification of empty spaces)

A spatio-temporal topology validator for the property units is implemented in Python (https://www.python.org/), version 2.7, using the ArcPy package for ArcGIS Desktop 10.3 (http://desktop.arcgis.com/en/arcmap/10.3/analyze/arcpy/what-is-arcpy-.htm). One main requirement is that property unit polygons should not overlap in space and time, that is, two polygons that cover the same area should not simultaneously exist. A pseudo code for this topology check is as follows:

For polygon i1, polygon i2

Check IF (i1 overlaps i2

OR i1 contains i2

OR i1 is within i2)

AND i1.eDate >= i2.sDate

AND i1.sDate <= i2.eDate

The ‘sDate’ and ‘eDate’ represent the start and end year; thus, the topology is evaluated for each year. Polygons that overlap are corrected by either re-evaluating their object-lifelines or adjusting their borders. The former is performed when errors are observed in the estimations of the start and end dates of the property units; the latter is conducted when minor overlaps are caused by non-perfect geometries.

The study area should not contain empty spaces, which appear when one polygon ceases to exist before another overlapping polygon starts to exist. To fill these empty spaces when no digitized geometries are available for a specific time period, new polygons are created by a subtraction method^7^. A total of 107 of the 1,159 property unit polygons were created in this manner.

## Additional Information

**How to cite this article:** Hedefalk, F. *et al.* Spatiotemporal historical datasets at micro-level for geocoded individuals in five Swedish parishes, 1813–1914. *Sci. Data* 4:170046 doi: 10.1038/sdata.2017.46 (2017).

**Publisher’s note:** Springer Nature remains neutral with regard to jurisdictional claims in published maps and institutional affiliations.

## Supplementary Material



## Figures and Tables

**Table 1:** Number of map documents digitized from historical maps.

**Map series**	**Years**	**No. of map documents**	**Scale**	**Digitized objects**
Land Survey Maps (LSMs)	1757–1863	39	1:4,000–1:8,000	Property units, buildings
Military Topographical Survey Maps (MTSMs)	1812–1820	11	1:20,000	Roads, buildings, streams, wetlands, lakes
Topographic Maps (TMs)	1860–1865	2	1:100,000	Roads, buildings
Economic Maps (EMs)	1910–1915	7	1:20,000	Property units, buildings, roads, railroads,
Cadastral Dossiers (CDs)	1757–1914	Approximately 150	1:1,000–1:8,000	Property units

**Table 2:** Overview of the datasets.

**Layer**	**Dataset name**	**Geometry**	**Time representation**	**No. of objects**
Property Units	property_units_SEDD.shp	Polygon	Object-lifelines	1,165
Wetlands	Wetlands.shp	Polygon	Object-lifelines	MTSM: 364Modern: 195
Buildings	Buildings_polygons.shp	Polygon	Snapshots	LSM: 385MTSM: 438
Buildings	Buildings_points.shp	Point	Snapshots	TM: 638EM: 4,216
Roads	Roads.shp	Line	Snapshots	MTSM: 839TM: 433EM: 2,060
Railroads	Railroads.shp	Line	Snapshots	EM: 150
Streams	Streams.shp	Line	Snapshots	MTSM: 99
Lakes	Lakes.shp	Polygon	Snapshots	MTSM: 40
‘No. of objects’ represents the number of geographic objects such as building points or road segments.				

**Table 3:** Property Units (property_units_SEDD.shp).

**Attributes**	**Type**	**Description**
FID	Integer	Shapefile automatic identifier
Shape	Geometry	Geometry of the object (polygon).
PolygonId	String	Unique identifier of the polygon. Created by the authors.
PuId	String	Property unit identifier created by the authors. This identifier establishes links on the property unit level between the geographic data and the individuals in the SEDD (cf. section ‘Links to the Scanian Economic Demographic Database (SEDD)’.
AuId	String	Address unit identifier created by the authors. This identifier establishes links on the address unit level between the geographic data and the individuals in the SEDD (cf. section ‘Links to the Scanian Economic Demographic Database (SEDD)’.
Parish	String	The parish that the property unit belongs to.
addrName	String	Address name (e.g., ‘Hög’).
addrCode	String	Address number (e.g., 04).
addrLetter	String	Address part (e.g., ‘B’ or ‘1’).
sDate	String	Start date of the object (usually only the year).
eDate	String	End date of the object (usually only the year).
sDateMin	String	The earliest start date (usually only the year), which represents an uncertainty interval of the object’s lifeline. That is, the object may exist as early as indicated in this attribute.
eDateMax	String	The latest end date (usually only the year), which represents an uncertainty interval of the object’s lifeline. That is, the object may exist as late as indicated in this attribute.
obsDate	String	Date of observation (=date of the map from which the object was digitized) (usually only the year).
mapCode	String	Code of the historical map from which the object was digitized. From the Lantmäteriet historical map archive^17^.
mapName	String	Name of the historical map from which the object was digitized. From the Lantmäteriet historical map archive^17^.
mapSeries	String	Name of the map series of the historical map from which the object was digitized.
taxValue	Float	Taxation value of the property unit (Swedish: *Mantal*). A ratio value indicating the productivity of the unit.
propForm	String	Type of property formation that created or altered the property unit (In Swedish; e.g., *Hemmansklyvning*).
propFormEn	String	English translation of the propForm attribute (e.g., subdivision).
ownerFNObs	String	First name of the owner of the property unit when observed in the map.
ownerLNObs	String	Last name of the owner of the property unit when observed in the map.
Subtype	String	Type of property unit (e.g., croft).
notePU	String	Comments made about the property unit when it was digitized (sometimes a transcription of a text part in the map document or cadastral dossier). This information is primarily relevant if the property units were to be improved or changed.
noteTimEst	String	Comments about the lifeline estimation of the property unit/object. This information is primarily relevant if the lifeline estimation were to be improved.
Shape_Leng	Float	Circumference of the property unit polygon (meters).
Shape_Area	Float	Area of the property unit polygon (square meters).
parishCode	String	National parish codes from Statistics Sweden (SCB).

**Table 4:** Wetlands in object-lifelines (Wetlands.shp).

**Attributes**	**Type**	**Description**
FID	Integer	Shapefile automatic identifier.
Shape	Geometry	Geometry of the object (polygon).
wetlandId	String	Unique identifier of the wetland. The same identifier is assigned to those overlapping historical and modern wetland polygons that represent the same wetland (but with different geometric shapes because of, e.g., drainage). Created by the authors.
sDateMin	Integer	The earliest start date (usually only the year), which represents an uncertainty interval of the object’s lifeline. That is, the object may exist as early as indicated in this attribute.
sDate	Integer	Specific start date (usually only the year). Almost always used when a joint drainage unit explicitly determines the start date of a modern wetland. E.g., a wetland was partially drained in 1889, and an overlapping, smaller, wetland can be observed in the modern data, this wetland is assigned the start date of 1890.That is, we assume that at this point in time, the modern wetland got its present geometric shape.The value ‘9999’ indicates that the wetland start within the interval that is defined by the sDateMin and sDateMax.
sDateAvg	Integer	An approximate start date for the wetlands observed in the modern data (usually only the year). Calculated as sDateMin+(eDateMin−sDateMin)/2. E.g., if a wetland observed in the modern data can earliest start in 1821, and earliest end at 2007 (the year it was digitized), its sDateAvg=1914.
sDateMax	Integer	The latest possible start date. Most often the year when the wetland was observed in the map.
eDateMin	Integer	The earliest possible end date (usually only the year), which represents an uncertainty interval of the object’s lifeline. That is, the object exists to at least the date indicated in this attribute. This date correspond often to the observation in the map. E.g., if a wetland was digitized from the MTSM map in 1820, eDateMin is set to 1820.
eDate	Integer	A specific date which most often indicate the year when a joint drainage was carried out on the wetland (usually only the year). The value ‘9999’ means that no joint drainage has been observed and that the wetland therefore stops to exist within the interval eDateMin and eDateMax.
eDateAvg	Integer	Used mostly for the historical wetlands observed in the 1820 MTSM map (usually only the year). Calculated as: sDateMax+(eDateMin−sDateMax)/2. If eDate is a specific date, eDateAvg is assigned the value of eDate.
eDateMax	Integer	The latest possible end date (usually only the year). The value ‘9999’ is used for the wetlands observed in the modern data and means ‘and onwards’ (i.e., it still exist and we do not know when it will stop existing).
obsDate	Integer	Date of observation (=date of the map from which the wetland was digitized) (usually only the year).
Source	String	The source of the wetland object.
wLandType	String	Type of wetland (only for wetlands observed in the modern data). Marshy or Very marshy.
Note	String	Note about the wetland object. This information is mainly relevant if the lifeline estimation were to be improved.
drainName	String	Name of the joint drainage related to the wetland.

**Table 5:** Buildings (Buildings_polygons.shp, Buildings_points.shp).

**Attributes**	**Type**	**Description**
FID	Integer	Shapefile automatic identifier.
Shape	Geometry	Geometry of the object (polygon/point).
Parish	String	The parish that the building is located in.
noteBuild	String	Note about the building.
addrName	String	Address name (e.g., ‘Hög’, or ‘Kågeröd church’).
addrCode	String	Address number (e.g., 04).
addrLetter	String	Address part (e.g., ‘B’ or ‘1’).
obsDate	String	Date of observation (=date of the map from which the object was digitized) (usually only the year).
mapCode	String	Code of the historical map from which the building was digitized. From the Lantmäteriet historical map archive^17^.
mapName	String	Name of the historical map from which the building was digitized. From the Lantmäteriet historical map archive^17^.
mapSeries	String	Name of the map series of the historical map from which the building was digitized.
subType	String	Type of building (e.g., church).
nameOnMap	String	Name or number on the historical map.
buildingId	String	Unique identifier of the building. Created by the authors.
parishCode	String	National parish codes from Statistics Sweden (SCB).

**Table 6:** Roads and railroads (Roads.shp, Railroads.shp).

**Attributes**	**Type**	**Description**
FID	Integer	Shapefile automatic identifier.
Shape	Geometry	Geometry of the object (line).
obsDate	String	Date of observation (=date of the map from which the object was digitized) (usually only the year).
mapCode	String	Code of the historical map from which the object was digitized. From the Lantmäteriet historical map archive^17^.
mapName	String	Name of the historical map from which the object was digitized. From the Lantmäteriet historical map archive^17^.
mapSeries	String	Name of the map series of the historical map from which the object was digitized.
subType	String	Type of road (e.g., passage way, regional way) or railroad (undefined).
roadId/railId	String	Unique identifier of the road/railroad segment. Created by the authors.
noteRoad/noteRail	String	Note about the road/railroad.

**Table 7:** Streams and lakes (Streams.shp, Lakes.shp).

**Attributes**	**Type**	**Description**
FID	Integer	Shapefile automatic identifier.
Shape	Geometry	Geometry of the object (line or polygon).
lakeId/streamId	String	Unique identifier of the lake or stream. Created by the authors.
obsDate	String	Date of observation (=date of the map from which the object was digitized) (usually only the year).
Name	String	Name of the object.
mapCode	String	Code of the historical map from which the object was digitized. From the Lantmäteriet historical map archive^17^.
mapName	String	Name of the historical map from which the object was digitized. From the Lantmäteriet historical map archive^17^.
mapSeries	String	Name of the map series from which the object was digitized.
subType	String	Type of lake/stream.
noteStream/noteLake	String	Note about the stream/lake.
Shape_Leng	Float	Length of the stream segment/circumference of the lake polygon (in meters).
Shape_Area	Float	Area of the lake polygon (in square meters) (only for Lake.shp).

**Table 8:** Positional accuracy—property unit centroids.

**RMSE**	**95% RMSE**	**Median error**	**s.d.**	**Min**	**Max**	**95th perc**
16.7	14.4	10.3	11.4	1.0	59.4	40.1

**Table 9:** Match rates for each geocoding level and parish.

**Geocoding level**	**Period**	**Persons**	**Person-years**	**Match rate (%)**	
				**Property unit**	**Address**
Hög	1813–1848	1,398	9,108	87.8	97.5
	1849–1914	3,408	33,035	83.8	97.9
Kävlinge	1813–1848	1,898	11,272	71.5	96.3
	1849–1914	9,507	66,486	63.2	91.7
Sireköpinge	1813–1848	2,388	18,337	0*	86.9*
	1849–1914	10,353	80,500	62.5	96.4
Halmstad	1813–1848	2,427	18,833	58.8	75.21
	1849–1914	6,806	56,397	54.8	94.9
Kågeröd	1813–1848	5,165	55,997	27.1	31.2
	1849–1914	10,850	10,3175	76.9	94.2
All parishes	1813–1848	12,826†	113,548	37.3‡	45.4‡
	1849–1914	39,350†	339,595	66.6	92.2
The match rate represents the percent of geocoded person-years. *Persons* represents the number of individuals who have lived within the specified parish.					

*The land reforms had not been conducted in Sireköpinge for the period 1813–1848. The individuals can be geocoded on the address level with the given match rate because most individuals lived within the village and did not live in their property units.

^†^The total number of individuals in this dataset for the period 1813–1914 is approximately 45,000, which is less than the 53,000 individuals mentioned in ref. 7. The reason is that the latter number includes all individuals with at least one event registered, whereas the former and publicly open dataset excluded individuals with only one event registered (individuals with only a birth, a death or a migration registered).

^‡^These match rates are low because some of the land reforms had not been implemented in this period. Thus, people still lived within the villages and cultivated nearby scattered plots.
